# Dental tissue fractures and their relationship with deciduous molar hypomineralization, molar incisor hypomineralization, and psychosomatic factors: a cross-sectional study

**DOI:** 10.1590/1807-3107bor-2026.vol40.001

**Published:** 2026-03-06

**Authors:** Jaine Viviane SILVA, Lucas de Lima Oliveira BARBOSA, Isabel Cristina Celerino de Moraes PORTO, Valdeci Elias dos SANTOS

**Affiliations:** (a) Universidade Federal de Alagoas – UFAL, Department of Pediatric Dentistry, Maceió, Alagoas, Brazil.

**Keywords:** Molar Hypomineralization, Dental Caries, Tooth Wear, Bruxism, Temporomandibular Joint Dysfunction Syndrome, Anxiety

## Abstract

This study aimed to investigate the relationship between post-eruptive dental fractures, hypomineralization, and psychosomatic factors such as anxiety, headache, awake bruxism, and temporomandibular disorder (TMD) in children aged 7 to 12 years. A cross-sectional study involving 274 children was conducted using clinical examinations based on the European Academy of Pediatric Dentistry (EAPD) criteria for the diagnosis of hypomineralization and post-eruptive breakdown. Structured and validated questionnaires were administered through interviews to assess psychosomatic factors such as anxiety, headache, awake bruxism, and TMD. Statistical analysis included descriptive and inferential analyses using Fisher’s exact test and the chi-square test. The margin of error was 5%, with a 95% confidence interval. Tissue breakdown was observed in 6.2% of children, with a higher prevalence in posterior teeth. A significant association was found between tissue breakdown and hypomineralization in deciduous (p=0.002) and permanent teeth (p < 0.001), especially when hypomineralization presented as yellow-brown (p < 0.001). Anxiety, awake bruxism, and TMD were not significantly associated with such fractures, but they were significantly associated with headache (p = 0.023). Additionally, dental caries (p = 0.003) and occlusal wear (p < 0.001) were associated with tissue breakdown. Post-eruptive breakages were found to be associated with dental enamel hypomineralization in deciduous and permanent teeth. Furthermore, dental caries and occlusal wear were identified as key contributing factors for these fractures. In conclusion, post-eruptive fractures were associated with enamel hypomineralization in both deciduous and permanent teeth, as well as with dental caries and occlusal wear. Among psychosomatic factors, only headache showed a significant association.

## Introduction

Hypomineralization is a qualitative developmental defect of enamel that occurs during its formation and maturation, making both enamel and dentin more porous and less mineralized.^1^ This condition can affect both deciduous and permanent teeth, and it is referred to as deciduous molar hypomineralization (DMH) and molar incisor hypomineralization (MIH), respectively.^2,3^ This developmental disorder of the tooth structure predisposes affected teeth to advanced carious lesions, occlusal wear, and post-eruptive tooth fractures.^4^ Furthermore, these predisposing factors are interrelated, precluding an analysis of their cause and effect.

In this context, bruxism-related behaviors such as clenching or teeth grinding — conditions closely associated with stress and anxiety — may contribute to a higher incidence of tissue fractures, particularly in teeth already affected by structural defects (e.g., hypomineralization).^5,6^ Nevertheless, a scientific gap persists concerning the role of awake bruxism in tissue damage in the oral cavity.

Of note, childhood anxiety is a known triggering factor for both bruxism and TMD,^7^ conditions that have been relatively understudied in pediatric populations.^7,8^ According to a recent meta-analysis, children with bruxism are 2.97 times more likely to have temporomandibular disorders (TMD). TMD encompasses a spectrum of musculoskeletal and neuromuscular conditions involving the masticatory muscles, temporomandibular joint (TMJ), and associated oral structures, with a strong relationship to psychological factors.^9^

The aim of this study was to examine the relationship between post-eruptive tooth fracture, developmental defects of enamel (hypomineralization), and psychosomatic factors such as anxiety, awake bruxism, and TMD. The study hypothesized that post-eruptive tooth fractures are associated with psychosomatic factors, including anxiety, headache, awake bruxism, TMD, and dental caries.

## Methods

This study was approved by the Research Ethics Committee of the Federal University of Alagoas (UFAL) (process no. 6.668.063). The research was conducted in accordance with the Declaration of Helsinki of the World Medical Association. Written consent was obtained from parents and/or guardians via an informed consent form, and assent was obtained from the participating children using an informed assent form. This research followed the Strengthening the Reporting of Observational Studies in Epidemiology (STROBE) guidelines.

This was a cross-sectional study using a simple random sampling method. The sample size was calculated using Epi Info^TM^ software (Centers for Disease Control and Prevention), considering a 23.3% prevalence of enamel and/or dentin fracture in children attending public schools in Maceió,^10^ Brazil, with a 95% confidence interval and a 5% margin of error. Based on this calculation, a minimum of 274 children aged 7 to 12 years was required for the study. Participants were recruited from public school playgrounds in the city of Maceió, in the state of Alagoas, northeastern Brazil. Clinical assessment and data collection took place between November 2023 and January 2024. Children with craniofacial and/or congenital syndromes or anomalies, neurological disorders, joint diseases, and cognitive impairments that could hinder cooperation during physical examination or comprehension of the questionnaires were excluded.

Data were collected through interviews with the children. Thus, the following conditions were assessed: awake bruxism,^5,11^ TMD, musculoskeletal symptoms,^12^ and anxiety levels, evaluated using the State-Trait Anxiety Inventory for Children.^13^

After conducting interviews to assess anxiety traits, children who achieved a minimum score of 31 points were classified as anxious, according to Bouden’s criteria.^13^ Regarding TMD, schoolchildren who reported pain in the masticatory structures and temporal headache — exacerbated by movement, function, or mandibular parafunction – and who exhibited tenderness upon palpation of the temporalis muscle were identified as having TMD. Finally, in the assessment of awake bruxism, children who answered positively to questions about grinding and/or clenching their teeth — excluding sleep-related episodes — were considered to have bruxism.

The intraoral examination was conducted first to minimize potential discomfort from palpation and to ensure the child’s comfort during the oral assessment. The examination was performed by a single researcher under natural lighting in the schoolyard, using a mouth mirror, spatula, and gauze pads. All children brushed their teeth prior to the examination. Occlusal wear was assessed based on the following clinical signs: attritional wear facets; enamel chipping, cracking, and/or fractures; and the presence of linea alba on the buccal mucosa or lateral borders of the tongue.^5^Tooth wear was classified according to the presence of enamel wear, visible dentin exposure, and loss of clinical crown height.^14^ Children with at least one of these clinical features were categorized as having occlusal wear.

Hypomineralization was clinically diagnosed, following the European Academy of Pediatric Dentistry (EAPD) criteria^15, 16^for MIH and DMH. Each tooth surface was scored as follows: normal (N), demarcated opacity (DO), post-eruptive enamel breakdown (PEB-E), post-eruptive dentin breakdown (PEB-D), atypical restoration (AR), or extracted (EXT).

If a tooth surface exhibited more than one characteristic of MIH or DMH, the following hierarchical classification was applied: extraction due to MIH or DMH > atypical restoration > post-eruptive fracture with dentin exposure > post-eruptive enamel fracture > yellow-brown opacity > creamy-white opacity. In cases of uncertainty regarding the severity of MIH or DMH, the examiner was instructed to classify the surface at the less severe level.

To identify post-eruptive fractures associated with prior hypomineralization, the following variables were combined: PEB-E, PEB-D, atypical restoration (AR), and EXT in both primary and permanent dentitions. The clinical examination for caries detection was conducted in accordance with the World Health Organization guidelines.^17^

A single examiner was calibrated for the diagnosis of MIH and DMH, yielding a high intra-examiner agreement, with kappa coefficients of 0.92 and 0.94, respectively. After data collection and variable categorization, a database was created for statistical analysis using SPSS (Statistical Package for the Social Sciences) version 23. The normality of data distribution was assessed using the Kolmogorov-Smirnov test. Associations between categorical variables were tested using the chi-square test or Fisher’s exact test, as appropriate. The margin of error was set at 5%, with a 95% confidence interval.

## Results

A total of 274 male and female children were evaluated, with a mean age of 9.22±1.41 years. MIH was present in 14.6% of the children, with male children significantly more affected than females (p = 0.006). Among those with MIH, 6.9% had lesions only in posterior teeth, 3.6% in anterior teeth, and 4.1% in both. Because of the age range, only five children (1.8%) exhibited hypomineralization in primary (deciduous) teeth. The presence of hypomineralization in primary teeth was associated with a rate ratio (RR) of 7.68 (5.64 to 10.46) for the development of MIH. In addition to MIH, occlusal wear (p < 0.001) and dental caries (p = 0.006) were significantly associated with MIH. Notably, 60.6% of the children had at least one carious lesion. Regarding psychosomatic factors, only awake bruxism showed a statistically significant association (p = 0.012), while anxiety (p=0.587) and TMD (p = 0.392) were not significantly associated (Table 1).

**Table 1:** Molar incisor hypomineralization and its relationship with dental problems and psychosomatic variables (anxiety, awake bruxism, and TMD).

Variable	No (%)	Yes (%)	Total (%)	p-value	RR (95%CI)
Sex					
Female	125 (91.2)	12 (8.8)	137 (100)		1
Male	109 (79.5)	28 (20.5)	137 (100)	P^(1)^ =0.006*	2.33
Total	234 (85.4)	40 (14.6)	274 (100)		(1.23–4.39)
MDH					
No	234 (86.9)	35 (13.1)	269 (100)		1
Yes	0 (0)	5 (100)	5 (100)	P^(2)^ =0.001*	7.68
Total	234 (85.4)	40 (14.6)	274 (100)		(5.64–10.46)
Caries					
No	158 (89.7)	18 (10,3)	176 (100)		1
Yes	76 (77.5)	22 (22.5)	98 (100)	P^(1)^ =0.006*	2.19
Total	234 (85.4)	40 (14.6)	274 (100)		(1.23–3.88)
DO					
No	228 (87.6)	32 (12.4)	260 (100)		1
Yes	6 (42.8)	8 (57.2)	14 (100)	P^(2)^ =0.001*	4.64
Total	234 (85.4)	40 (14.6)	274 (100)		(2.65– 8.10)
AB					
No	197 (87.9)	27 (12.1)	224 (100)		1
Yes	37 (74)	13 (26)	50 (100)	P^(1)^ =0.012*	2.15
Total	234 (85.4)	40 (14.6)	274 (100)		(1.20–3.87)
TMD					
No	225 (85.8)	37 (14.2)	262 (100)		1
Yes	9 (75)	3 (25)	12 (100)	P^(2)^ =0.297	1.77
Total	234 (85.4)	40 (14.6)	274 (100)		(0.63–4.93)
Anxiety					
No	178 (84.7)	32 (15.3)	210 (100)		1
Yes	56 (87.5)	8 (12.5)	64 (100)	P^(1)^ =0.587	0.82
Total	234 (85.4)	17 (14.6)	274 (100)		(0.39–1.68)

MIH: Molar incisor hypomineralization; MDH: Molar deciduous hypomineralization; DO: Occlusal wear; AB: Awake bruxism; TMD: Temporomandibular disorder. ^(1)^Using the chi-square test; ^(2)^Using Fisher’s exact test. *Significant association at 5.0%.

Tissue fractures were observed in 6.2% of the children, affecting either enamel alone or both enamel and dentin. Most of these fractures (95.6%) occurred in posterior teeth. Structural damage was significantly associated with hypomineralization in both primary (p = 0.002) and permanent teeth (p<0.001). When considering the color of hypomineralization, yellow-brown lesions were more prone to fracture (p < 0.001). The presence of tissue fracture was linked to an RR of 5.00 (1.31–18.96) for yellow-brown lesions compared to creamy-white ones. Dental caries (p = 0.003) and occlusal wear (p < 0.001) were also significantly associated with fractures. Awake bruxism (p = 0.096), anxiety (p = 0.986), and TMD (p = 0.544) were not statistically associated with tissue damage. Nevertheless, headache showed a significant association (p = 0.023). No statistically significant association was observed between sex and fracture, but age was a significant factor, with a higher prevalence of fractures in children aged 10 to 12 years (p < 0.001) (Table 2).

**Table 2:** Enamel fracture and its relationship with dental conditions and psychosomatic variables (anxiety, awake bruxism, and TMD).

Variable	No (%)	Yes (%)	Total (%)	p-value	RR (95%CI)
Sex					
Female	131 (95.6)	6 (4.4)	137 (100)		1
Male	126 (91.9)	11 (8.1)	137 (100)	P^(1)^ =0.211	1.83
Total	257 (93.7)	17 (6.3)	274 (100)		(0.69–4.81)
Age (years)					
7–10	204 (96.6)	7 (3.4)	211 (100)		1
11–12	53 (84.1)	10 (15.9)	63 (100)	P^(1)^ =0.001*	4.78
Total	257 (93.7)	17 (6.3)	274 (100)		(1.89–12.05)
MIH					
No	234 (100)	0 (0)	234 (100)		1
Yes	23 (57.5)	17 (42.5)	40 (100)	P^(2)^ =0.001*	11.1
Total	257 (93.7)	17 (6.3)	274 (100)		(7.56–16.5)
DMH					
No	255 (94.7)	14 (5.3)	269 (100)		1
Yes	2 (40)	3 (60)	5 (100)	P^(2)^ =0.002*	11.52
Total	257 (93.7)	17 (6.3)	274 (100)		(4.78–27.76)
Color					
No change	234 (100)	0 (0)	234 (100)		-
CWO	14 (87.5)	2 (12.5)	16 (100)	P^(2)^ =0.001*	1
YBO	9 (37.5)	15 (62.5)	24 (100)		5
Total	257 (93.7)	17 (6.3)	274 (100)		(1.31–18.96)
Caries					
No	107 (99)	1 (1)	108 (100)		1
Yes	150 (90.3)	16 (9.7)	166 (100)	P^(2)^ =0.003*	10.4
Total	257 (93.7)	17 (6.3)	274 (100)		(1.40–77.3)
DO					
No	249 (95.7)	11 (4.3)	260 (100)		1
Yes	8 (57.1)	6 (42.9)	14 (100)	P^(2)^ =0.001*	10.12
Total	257 (93.7)	17 (6.3)	274 (100)		(4.38–23.39)
AB					
No	213 (95)	11 (5)	224 (100)		1
Yes	44 (88)	6 (12)	50 (100)	P^(2)^ =0.096	2.44
Total	257 (93.7)	17 (6.3)	274 (100)		(0.94–6.29)
TMD					
No	246 (93.8)	16 (6.2)	262 (100)		1
Yes	11 (91.6)	1 (8.4)	12 (100)	P^(2)^ =0.544	1.36
Total	257 (93.7)	17 (6.3)	274 (100)		(0.19– 9.45)
Anxiety					
No	197 (93.8)	13 (6.2)	210 (100)		1
Yes	60 (93.7)	4 (6.3)	64 (100)	P^(1)^ =0.986	1
Total	257 (93.7)	17 (6.3)	274 (100)		(0.34– 2.98)
Headache					
No	175 (70.8)	82 (29.2)	247 (100)	P^(1)^ =0.023*	1
Yes	7 (41.1)	10 (58.9)	17 (100)		1.84
Total	182 (66.4)	92 (33.6)	274 (100)		(1.19–2.85)

MIH: Molar incisor hypomineralization; MDH: Molar deciduous hypomineralization; DO: Occlusal wear; AB: Awake bruxism; YBO- yellow-brown opacity; CWO- creamy-white opacity; Temporomandibular disorder; RR: rate radio. ^(1)^Using the chi-square test; ^(2)^Using Fisher’s exact test. *Significant association at 5.0%.

## Discussion

The global prevalence of MIH varies widely, ranging from 2.8% to 40.2%, with a mean prevalence of 13.5% worldwide.^18,19^ In the present study, 40 out of 274 schoolchildren were diagnosed with hypomineralization, representing 14.6% of the sample, slightly exceeding the global average. Male children were more frequently affected (p = 0.006). The literature, however, does not show a consistent pattern of sex-related differences. Sluka et al.^20^ and Hussein et al.^21^ reported no sex-related differences, Jeremias et al.^22^ observed a higher prevalence of hypomineralization among female children.

The presence of hypomineralization in the deciduous dentition was associated with an RR of 7.68 for the development of MIH. This finding aligns with those obtained by Elfrink et al.^23^, who reported that children with DMH were 4.4 times more likely to develop MIH.^23^ The presence of DMH has therefore been proposed as a predictor of hypomineralization in the permanent dentition, as the eruption of deciduous second molars precedes that of permanent molars. This association may be explained by the overlap in the developmental and mineralization stages of the affected teeth, although the maturation phase of permanent molars is considerably longer.^23^

Hypomineralized enamel exhibits inferior mechanical properties compared to healthy enamel.^24,25^It is characterized by elevated carbon (C) content and reduced levels of phosphorus (P) and calcium (Ca), resulting in lower hardness and elastic modulus.^24-26^ Additionally, hypomineralized enamel contains high protein concentrations that inhibit hydroxyapatite crystal growth and interfere with enzymatic activity during enamel maturation.^26^ As a result, MIH-affected teeth display structural porosities, making them more susceptible to dental caries and post-eruptive fractures.^2^A statistically significant association was observed between MIH and caries, with the RR of caries in hypomineralized teeth approximately twice that of healthy teeth.

Post-eruptive breakdown in hypomineralized teeth was detected in both deciduous and permanent teeth, with an RR 11 times higher than in unaffected teeth. Post-eruptive breakdown was predominantly observed in posterior teeth (95%), which likely bear the greatest masticatory forces.^27, 28^ Consequently, it is essential that such teeth be monitored by trained dental professionals, who can implement preventive measures based on clinical indicators such as enamel and/or dentin fractures, atypical restorations, well-demarcated opacities, and variations in lesion color and extent.^15, 16^

Jälevik and Norén^29^ concluded that teeth with demarcated yellow-brown opacities are at greater risk of post-eruptive fracture compared to those with creamy-white lesions. This finding is supported histologically by increased enamel porosity, reduced mineral content, and elevated organic content in the more intensely colored lesions.^29,30^ Consistent with these findings, the RR of fracture in teeth with yellow-brown hypomineralization was five times higher than in teeth with creamy-white lesions. Therefore, the color of MIH lesions plays a significant role in the likelihood of enamel and/or dentin fracture.

This study also found a statistically significant association between dental caries and post-eruptive fracture (p = 0.003). Considering that dental caries is a risk factor for the development of such fractures, the RR of fracture in the presence of caries was 10 times higher than in caries-free teeth. Nonetheless, this relationship is bidirectional, as fractures can lead to cavity formation, which, in turn, facilitates caries development.^31^ Additionally, hypomineralized lesions may predispose teeth to caries, mechanically weakening the tooth structure and resulting in post-eruptive fractures.^32^

In addition, children in the older age group (10 to 12 years) accounted for the majority of post-eruptive fractures (Table 2). This trend may be attributed to the increased porosity and demineralization associated with MIH,^33^ increasing the vulnerability of affected teeth to occlusal wear and dental caries.^2^ The findings of this study revealed a statistically significant association between occlusal wear and post-eruptive tooth fractures. Despite the presence of occlusal wear and clinical signs commonly linked to bruxism, no statistically significant association was found between enamel fractures and awake bruxism, although the RR of fracture in children with bruxism was 2.44 times higher than in those without bruxism.

Headache was also significantly associated with dental tissue fractures, indicating that children experiencing such fractures were 1.84 times more likely to report facial or cranial pain. This supports the notion that hypomineralization and its consequences may negatively impact quality of life.^35^ A recent systematic review by Jawdekar et al.^34^ found that children with MIH are 17 and 25 times more likely to experience quality-of-life impairments, particularly due to facial pain and headache, which is often linked to caries, tissue fractures, and the need for restoration replacement.^27^ Therefore, the findings of the present study are consistent with those described in the existing literature.

## Conclusion

Post-eruptive fractures were significantly associated with enamel hypomineralization in both primary and permanent teeth. In addition, dental caries and occlusal wear were identified as contributing risk factors for these fractures. No significant associations were found between psychosomatic factors and post-eruptive breakdown, although headache was notably associated with these events.

## Data Availability

The content will be available when the article is published.
